# Fine-Scale Risk Mapping for Dengue Vector Using Spatial Downscaling in Intra-Urban Areas of Guangzhou, China

**DOI:** 10.3390/insects16070661

**Published:** 2025-06-25

**Authors:** Yunpeng Shen, Zhoupeng Ren, Junfu Fan, Jianpeng Xiao, Yingtao Zhang, Xiaobo Liu

**Affiliations:** 1 School of Civil Engineering and Geomatics, Shandong University of Technology, Zibo 255000, China; 22407010006@stumail.sdut.edu.cn; 2 State Key Laboratory of Resources and Environmental Information System, Institute of Geographic Sciences and Natural Resources Research, Chinese Academy of Sciences, Beijing 100101, China; 3 College of Resources and Environment, University of Chinese Academy of Sciences, Beijing 100049, China; 4 Guangdong Provincial Institute of Public Health, Guangzhou 511430, China; jpengx@163.com; 5 Guangdong Provincial Center for Disease Control and Prevention, Guangzhou 511430, China; zhangyt9@mail2.sysu.edu.cn; 6 National Key Laboratory of Intelligent Tracking and Forecasting for Infectious Diseases, National Institute for Communicable Disease Control and Prevention, Chinese Center for Disease Control and Prevention, Beijing 102206, China; liuxiaobo@icdc.cn; 7 Department of Vector Control, School of Public Health, Cheeloo College of Medicine, Shandong University, Jinan 250012, China

**Keywords:** vector surveillance and control, mosquito risk maps, spatial downscaling, data resampling

## Abstract

Generating fine-scale risk maps for mosquito-borne diseases vectors is an essential tool for guiding spatially targeted vector control interventions in urban settings, given the limited public health resources. This study aimed to generate fine-scale risk maps for dengue vectors using routine vector surveillance data collected at the township scale. We integrated monthly township-specific Breteau Index (BI) data from Guangzhou city (2019 to 2020) with covariates extracted from remote sensing imagery and other geospatial datasets to develop an original random forest (RF) model for predicting hotspot areas (BI ≥ 5). We implemented three data resampling techniques (undersampling, oversampling, and hybrid sampling) to improve the model’s performance and evaluate it using the ROC-AUC, Recall, Specificity, and G-means metrics. Finally, we generated a downscaled risk maps for BI hotspot areas at a 1000 m grid scale by applying the optimal model to fine-scale input data. Our findings indicate the following: (1) data resampling techniques significantly improved the prediction accuracy of the original RF model, demonstrating robust spatial downscaling capabilities for fine-scale grids; (2) the spatial distribution of BI hotspot areas within townships exhibits significant heterogeneity. The fine-scale risk mapping approach overcomes the limitations of previous coarse-scale risk maps and provides critical evidence for policymakers to better understand the distribution of BI hotspot areas, facilitating pixel-level spatially targeted vector control interventions in intra-urban areas.

## Simple Summary

Routine mosquito surveillance data and risk maps are often generated on coarse spatial scales (i.e., counties and townships), making it challenging to accurately locate hotspots and risk areas of high infestation within administrative areas. This study aimed to downscale mosquito risk maps from township scale to kilometer grid scale using spatial downscaling techniques. Our findings indicate the following: (1) the data resampling technique significantly improves the predictive accuracy of the original random forest model for predicting hotspot areas, demonstrating robust spatial downscaling capabilities in generating fine-scale risk maps; (2) the hotspot areas of dengue vectors within townships are not uniformly distributed and exhibit substantial heterogeneity. The fine-scale risk maps generated through downscaling techniques from coarse scale provide accurate spatial information and scientific evidence for implementing spatially targeted vector control interventions in hotspot areas.

## 1. Introduction

*Aedes albopictus* plays a crucial role in the transmission of several mosquito-borne diseases, including dengue fever (DF), Zika virus, and yellow fever, posing a significant challenge to global public health [1,2,3]. A previous study shows that *Ae. albopictus* prefer peri-domestic habitats, laying eggs in artificial water storage containers [4]. In China, community-involved vector control aiming at reducing larval density remains the primary strategy for interrupting the transmission chain of DF, especially in the absence of effective vaccines or drugs [5,6]. However, existing routine surveillance data and mosquito risk maps are generally characterized by coarse spatial scales (e.g., townships and counties), which makes it difficult to quickly locate hotspot areas and risk areas of high infestation [7,8,9]. In this context, generating a fine-scale risk map for dengue vectors hotspot areas is important for facilitating spatially targeted vector control, and improving the cost-effectiveness of mosquito prevention efforts.

The routinely collected *Ae. albopictus* larval data is an important foundation for assessing local mosquito density levels and delineating hotspot areas [10]. Various entomological indices (e.g., House Index (HI), Container Index (CI), and Breteau Index (BI)) are commonly used to quantify vector density, especially the BI (the number of positive containers per 100 households inspected) [11]. Previous studies show that BI is more sensitive to the changes in the number of houses and containers under inspection, is more convenient, and can obtain high accuracy in low-density areas compared with other larval indices [12,13]. The BI has been widely adopted for dengue vector control in China and other endemic regions [13]. In additional, some studies have applied Geographic Information System (GIS) tools to predict potential hotspots and generating risk maps [12,14,15]. These risk maps also align with the needs of decision-makers in guiding vector surveillance, as the goal of monitoring is to prioritize reducing mosquito density in hotspot areas, rather than eliminate mosquitoes completely [1,14,16].

Mosquito risk mapping techniques have shifted from field data to automated predictive models in recent years [17]. These predictive methods of generating mosquito risk maps by integrating environmental variables and vector surveillance data have been validated in both rural and urban areas with high performance [7,18,19]. However, a notable limitation of these risk maps is coarse spatial resolution, which limits their potential for practical application in vector surveillance [20]. To overcome this, several studies have proposed using field data with detailed spatial information, rather than relying on aggregated data at the administrative level, to generate fine-scale risk maps [21,22,23]. For example, in [1,24] the authors collected field mosquito surveillance data and environmental data to generate a risk map with a resolution of 5000 m and 250 m. However, these methods also face challenges in obtaining detailed geographic coordinates for each surveillance sites, including China [23]. Fortunately, recent research suggests spatial downscaling methods may be an alternative approach for generating fine-scale risk maps, even if fine-scale vector surveillance data are unavailable [25,26].

Downscaling mosquito surveillance data is essential for guiding public health departments in implementing spatially targeted vector control efforts. It has been widely applied in mosquito-borne diseases research to generate fine-scale risk maps [26,27]. High-resolution covariate data and robust predictive models are key factors influencing the accuracy of spatial downscaling. In this study, we extract auxiliary variables from remote sensing imagery and geographic data with a spatial resolution of ≤1000 m. Additionally, we choose the random forest (RF) model, which has proven advantages in handling multicollinearity and minimizing overfitting compared to other models [28,29,30], as the base prediction model. Meantime, we use data resampling methods to optimize the performance of the RF model [31]. Finally, we will then use the optimal RF model to downscale BI hotspot areas from township scale to fine-scale (1 km × 1 km) and generate risk maps.

In conclusion, to refine the spatial scale of existing mosquito risk maps and characterize the spatial heterogeneity of hotspot areas within townships, this study employed a spatial downscaling method to generate finer-scale risk maps, using the city of Guangzhou as a case study. The spatial downscaling method fills the research gap of directly using aggregated vector surveillance data (BI) to generate fine-scale risk maps, and characterizes the spatial heterogeneity of hotspot areas in intra-urban areas. These fine-scale mosquito risk maps provide key evidence for local policymakers to optimize current vector surveillance programs and offer fine-scale spatial information to prioritize spatially targeted vector control efforts in hotspot areas.

## 2. Materials and Methods

### 2.1. Study Area

**Figure 1:**
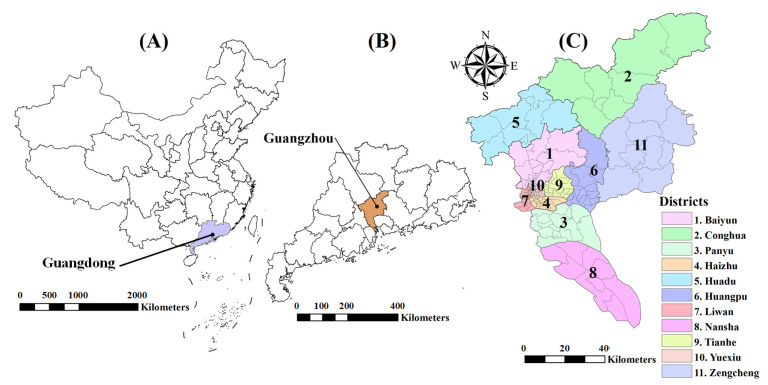
Study area for Guangzhou city. (**A**) shows the Guangdong Province in China; (**B**) shows the Guangzhou city in Guangdong province; (**C**) shows the locations of administrative districts in Guangzhou city.

Guangzhou is located between 112°57′ and 114°03′ E and between 22°26′ and 23°56′ N, covering an area of approximately 7434 square kilometers. The city consists of 11 districts and 167 townships (Figure 1). Guangzhou experiences a typical subtropical monsoon climate, characterized by high temperatures, rainy summers, and mild, humid winters. The annual average temperature of approximately 22 °C and precipitation of about 1736 mm [32]. These climatic conditions provide a suitable environment for the growth of dengue vectors, *Ae. albopictus* [33].

Additionally, since 1978, Guangzhou has faced significant challenges in DF prevention and control. The city experienced three large-scale outbreaks with over 1000 cases in 2006, 2013, and 2014, which accounted for more than 80% of the total cases in China [4,34]. The heavy disease burden of DF and favorable environmental conditions for larval growth make Guangzhou an ideal study area to explore the high-risk (hotspot) area mapping of dengue vectors on a finer scale.

### 2.2. Entomological Data

**Figure 2:**
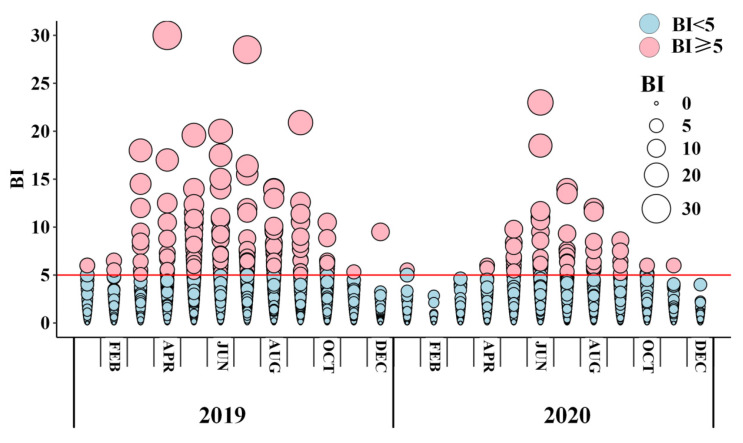
Monthly distribution of BI from 2019 to 2020. Each circle represents the BI value for a township and the red line (BI = 5) indicates the reference threshold for vector control and risk managements.

According to the guidelines issued by the Chinese Center for Disease Control and Prevention (GB/T 23797-2020) [35], routine vector surveillance for *Ae. albopictus* is conducted once or twice a month in Guangzhou. The monitoring content usually includes the collection of larvae or eggs from more than 100 households from eight kinds of potential breeding sites (including residential areas, parks, schools, etc.). Containers confirmed by professionals to contain *Ae. albopictus* will be regarded as positive containers and used to calculate the BI in combination with the number of households, as shown in Formula 1. In addition, due to the lack of accurate location information of field monitoring, the BI provided by the Guangdong Provincial Center for Disease Control and Prevention are aggregated at the township level. According to statistics, the total number of households surveyed in 2019–2020 exceeded 518,478 [36]. Finally, the original survey data is used to calculate the monthly average BI value of each townships and represent the local *Ae. albopictus* abundance. Figure 2 illustrates the vector surveillance data over the entire study period from 2019 to 2020.

(1) $$Breteau\;Index(BI)=\frac{Number\;of\;positive\;containers}{Number\;of\;houses\;inspected}\times 100$$

### 2.3. Environmental and Socio-Economic Data

#### 2.3.1. Meteorological Data

Climate factors have a significant correlation with the habitats suitable for larval distribution and growth rate, particularly rainfall and temperature [37]. In this study, we selected the monthly average temperature and monthly cumulative rainfall dataset, with a spatial resolution of 1 km, published by [38], as the primary meteorological data (Table 1). This dataset utilized the Delta spatial downscaling method to derive fine-scale meteorological data for the entire region of China by downscaling global climate datasets from CRU and WorldClim. Finally, we used the ArcGIS10.6 (ESRI, Redland, CA, USA) software program to extract the average temperature and cumulative rainfall for each townships and 1000 m grids from this dataset.

#### 2.3.2. Vegetation Data

NDVI, a proxy measure of vegetation, serves as an essential indicator to measure the habitat quality of larval growth [29]. High vegetation can shield the ground from direct sunlight and create a more humid environment conducive to the growth of larvae. We collected Sentinel-2 imagery (spatial resolution: 10 m; temporal resolution: 15 days) for Guangzhou from 2019 to 2020 using the Google Earth Engine (Table 1). Additionally, to address the issues that pixels miss caused by cloud cover, we employed a maximum value composite algorithm to fill in all missing pixels. Finally, we generated monthly NDVI maximum value images covering the entire area of Guangzhou city. The NDVI values range from −1 to 1, and higher values indicate greener vegetation. We also used the ArcGIS10.6 software program to extract the monthly average NDVI for each township and 1000-m grid scale.

#### 2.3.3. Land Use Data

Larval abundance and spatial distribution are strongly associated with land use [39]. Considering that land use is relatively stable in the short term, we chose the land use dataset released by Peking University for 2019 Guangzhou to represent the study period (Table 1). This dataset reclassifies land use into 12 classes, including farmland, urban areas, grasslands, forests, and other land use classes with a spatial resolution of 2.4 m [31]. The Shannon evenness index (SHEI) serves as a comprehensive measure for assessing the spatial uniformity of land use classes [31,39]. The SHEI value was extracted by the ArcGIS 10.6 software both for townships and 1000 m grid scale.

#### 2.3.4. Population Density Data

Population density is significantly correlated with larval densities and their spatial distribution [40]. Densely populated areas provide sufficient food sources for adult mosquitoes and containers for larval growth, significantly affecting the larval density and spatial distribution [10]. We used global population data with a resolution of 100 m published by WorldPop in 2020, to represent the population count throughout the study period (Table 1). Then, we used the ArcGIS 10.6 software program to summarize the population for each township and calculated the population density (people/km^2^) based on the areas of each administrative district. The data processing procedure for the 1000 m grid scale followed the same methodology as that for the township.

### 2.4. Methods for Data Analysis

#### 2.4.1. Variables Selection

We followed a three-step process to select a set of explanatory variables for the predictive model [41]. First, we identified environmental variables of significant biological importance for *Ae. albopictus* larvae growth based on relevant studies in the literature [42,43]. Next, we conducted a univariate analysis to select variables that improve model predictive performance. A variable was removed if its AUC value was below 0.5, indicating that it did not contribute meaningfully to the improvement of the model. Finally, we calculated Pearson’s correlation coefficients and Spearman’s rank correlation coefficients among the variables, discarding those with correlations greater than 0.75 to mitigate multicollinearity [44]. Once the univariate and collinearity analyses were satisfied, we prioritized selected the variables with higher univariate predictive capability (i.e., higher AUC values).

#### 2.4.2. Data Imbalance and Data Resampling

Data imbalance is a common issue in infectious disease prediction and vector distribution modeling studies. It is typically characterized by a significant difference in sample sizes between different classes [45]. In this study, we used the World Health Organization’s recommended vector control threshold of BI = 5 to transform larval density into a binary variable (0 = non-hotspot areas; 1 = hotspot areas) [6]. The final dataset comprised 218 positive samples (hotspot areas) and 3223 negative samples (non-hotspot areas), yielding an imbalance ratio of 1:15, which is considered an imbalanced dataset [46]. In our study, we applied undersampling, oversampling, and hybrid sampling methods to the original dataset to generate a new balanced dataset. The undersampling method randomly reduces the number of majority class samples to match the numbers of minority class samples, while the oversampling method increases the number of minority class samples through random duplication to match the numbers of majority class samples. The hybrid sampling method combines both approaches by applying oversampling to the minority class and undersampling to the majority class, resulting in a balanced dataset.

We set a sampling ratio of *p* = 0.5 to ensure the resampled dataset achieves a balanced state. In this study, we used above data resampling methods to process approximately 80% of the training data from the original surveillance dataset. The specific characteristics of final balanced dataset are described as follows: (1) in the undersampling method, the sample size of the minority class is 167, accounting for about 49% of the total samples, and the sample size of the majority class is 177, accounting for about 51%; (2) in the oversampling method, the sample size of the minority class is 2575, accounting for about 50% of the total samples, and the sample size of the majority class is 2576, accounting for about 50%; (3) in the hybrid sampling method, the sample size of the minority class is 1416, accounting for about 51% of the total samples, and the sample size of the majority class is 1336, which accounts for about 49% of the total samples.

#### 2.4.3. Random Forest Model and Spatial Downscaling

Random forest (RF) is a classic ensemble model comprising multiple decision trees [47]. The parameters *mtry* and *n.trees* are critical parameters that influence the model’s performance [48]. *N.trees* determines the number of trees in the model, while *mtry* specifies the number of features utilized by each tree for prediction. Each decision tree makes predictions by randomly selecting *mtry* features, and the final prediction of the RF model is the average of the predictions from all decision trees. In this study, 80% of the original dataset was randomly selected for training and parameter tuning, while the remaining 20% was used for model validation and accuracy assessment. In addition, we also built a predictive model which used the first-year data (i.e., 2019) and was validated by the second-year data (i.e., 2020). A 10-fold cross-validation repeated was employed to tune model parameters and we found that when *mtry* = 2 and *n.trees* = 1000, the model’s accuracy remained stabilized. Additionally, to improve the predictive performance, we separately trained the RF model using three resampling approaches and selected the optimal models for downscaling the high-risk areas for BI from the township scale to the 1000 m grid scale. Finally, we summarized the average of the outcomes from 30 repeated experiments as the final prediction to minimize the random impact on the results. All RF models were implemented in R 4.3.3 using the ‘‘random forest’’ and ‘‘ROSE’’ packages.

The fundamental premise of spatial downscaling methods is based on the assumption that a detectable relationship exists between information across different spatial scales, allowing for the inference of fine-scale outputs from coarse spatial scale inputs [26]. Therefore, when fine-scale vector surveillance data is absent or unavailable, it is theoretically possible to obtain fine-scale spatial downscaling results by using pre-trained models with robust performance on large spatial scales, combined with fine-scale input data. Finally, we completed the spatial downscaling mapping of hotspot areas for BI at a 1000 m grid scale, based on the optimal RF model developed at the township scale and input data collected at a 1000 m grid scale.

#### 2.4.4. Model Evaluation

Predictive performance refers to the classifier’s capability to accurately distinguish BI categories, such as high-risk (hotspot) areas and low-risk areas (BI ≥ 5/BI < 5). We used the 10th percentile of the predicted result as a threshold to convert the continuous probability predictions into binary variables to assess the model’s accuracy in predicting hotspot areas, respectively [44]. Recall measures the model’s ability to correctly capture hotspot areas, while specificity quantifies its ability to correctly capture non-hotspot areas. Additionally, we also selected ROC-AUC and G-means metrics to evaluate the model’s overall predictive performance. The ROC curve is a graphical representation formed by connecting recall and 1-specificity across various classification thresholds [49]. The area under the ROC curve (AUC) provides a comprehensive assessment of the model’s performance. G-means aims to evaluate a model’s performance by calculating the geometric mean of recall and specificity at a specified classification threshold. Indeed, the G-means has an advantage in avoiding overfitting negative samples and mitigating the marginalization of positive samples [50]. All evaluation metrics range from 0 to 1, with higher values indicating higher accuracy [18,51,52]. Ideally, the optimal classifier should perform well across all metrics. However, when this condition is difficult to satisfy at the same time, the optimal model can be recognized in the following conditions [6]: (1) the model obtained the highest recall on BI hotspot areas if the specificity of non-hotspot areas for BI was above 50%, or (2) a minimal decline of recall but a significant increase in specificity.

We also conducted a series of sensitivity analyses to test the robustness of the model performance. We compared two models which used average rainfall and cumulative rainfall as predictors, monthly and biweekly vector surveillance data, and whether vector control treatments were incorporated.

## 3. Results

Based on the variable selection criteria, we selected monthly average temperature, monthly cumulative rainfall, monthly average NDVI, population density, and SHEI as explanatory variables for the RF models in predicting BI hotspot areas (BI ≥ 5) at the township level in Guangzhou, as illustrated in Figures S1–S3. Monthly cumulative rainfall, other than average rainfall, was selected because of the higher performance (Table S1). To identify the optimal model for downscaling the BI hotspot areas, we compared the performance of the original model and three data resampling predictive models (undersampling, oversampling, and hybrid sampling). Finally, the undersampling model exhibited superior performance in predicting BI hotspot areas and was subsequently employed to downscale the risk maps from a township scale to a 1000 m grid scale.

### 3.1. Comparing Model Performance

We compared the performance of four predictive models and selected the optimal model for spatial downscaling, as shown in Table 2. All models exhibited similar ROC-AUC values of approximately 0.84, significantly exceeding the random guessing baseline (AUC = 0.5). The recall for Model 2 achieved the highest at approximately 0.7977, an increase of approximately 42% to 223% compared to the other models. The specificity for Model 2 exhibited the lowest accuracy at approximately 0.7682, a decrease of approximately 0.1714 to 0.221 compared to the other models. Additionally, we also compared the G-means metric and found that Model 2 achieved the highest value of 0.7821. Given the strong correlation between the DF epidemic and BI hotspot areas, along with the more severe consequences of misclassifying BI hotspot areas, Model 2 was recognized as the optimal model.

Our results also indicate that there was no significant difference in the identification of the BI hotspot areas between the two validation methods which used the second-year data as the test data and 20% of the data used as test data (Table S2). The model which incorporated vector control treatments did not significantly improve the prediction accuracy of BI hotspot areas (Figure S4). In addition, there was no significant difference between the two models which used monthly and biweekly vector surveillance data to identifying the BI hotspot areas (Table S3).

### 3.2. Risk Mapping at Township Scale

The 10th percentile threshold was also employed to classify the continuous predicted probability from the undersampling model into two categories, BI ≥ 5 and BI < 5, thus generating risk maps. Regions classified as BI hotspot areas indicate these townships may have higher risk of DF outbreaks and higher probabilities for larval infestation. Conversely, regions with predicted probabilities below the 10th percentile were categorized as lower likelihood of larvae infestation and DF outbreaks.

**Figure 3:**
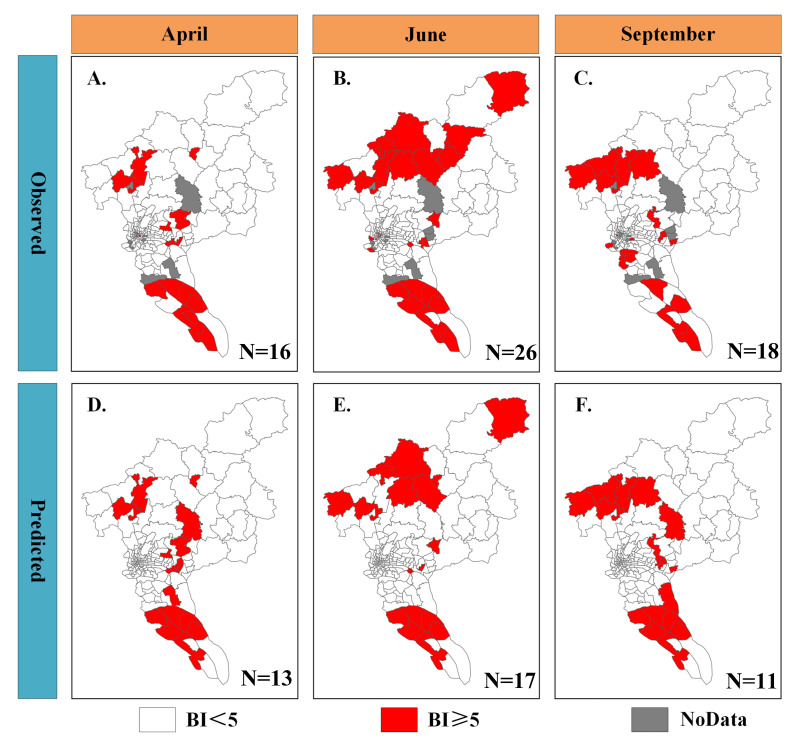
Mosquito risk maps both for observed and predicted results at the townships scale for Guangzhou in 2019. Panels (**A**–**C**) illustrate the spatial distribution of risk areas derived from BI surveillance data, with the numerical annotations indicating the count of high-risk (hotspot) townships. Panels (**D**–**F**) depict the spatial distribution of risk areas predicted by the optimized model, with the numerical annotations indicating the number of correctly predicted high-risk (hotspots) townships.

We used the optimal model to verify the spatial distribution consistency between the predicted and observed results at the township level during the mosquito active period in 2019, as shown in Figure 3. The analysis results indicated that the recall of the optimal model ranges from 0.6 to 0.8, demonstrating its robust capability in predicting BI hotspot areas. A comparative analysis of Figure 3A,D revealed that the predictive model correctly predicted 13 BI hotspot areas, yielding a recall of 81%. Similarly, a comparison of Figure 3B,E indicated that the model accurately predicted 17 BI hotspot areas, yielding a recall of 65%. A comparison of Figure 3C,F indicated that the predictive model accurately predicted 11 BI hotspot areas, yielding a recall of 61%. In addition, our prediction results also show that townships lacking vector monitoring data and areas with a low BI value in routine monitoring data may still be BI hotspot areas, as shown in Figure S5. These outstanding performance demonstrated the predictive model had a robust capability to downscale BI hotspot areas from township scale to a finer scale.

### 3.3. Risk Mapping at Township and Kilometer Grids Scale

**Figure 4:**
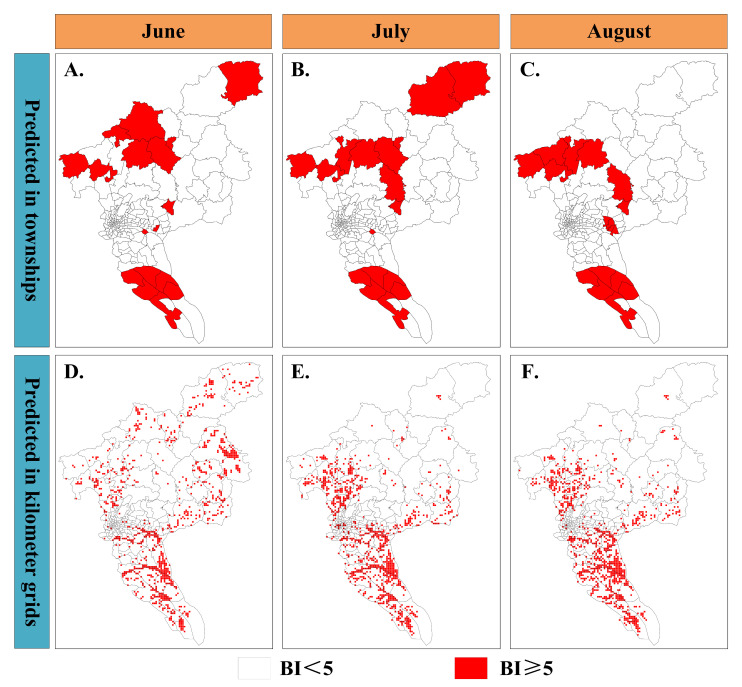
Mosquito risk maps both for township and 1000 m grid scale, 2019. Panels (**A**–**C**) illustrate the spatial distribution of BI hotspot areas on the township scale; Panels (**D**–**F**) show the spatial distribution of BI hotspot areas on the 1000 m grid scale.

We implemented spatial downscaling of BI hotspot areas at the township scale in Guangzhou using the optimal predict model and input data at a 1000 m grid scale. A 10th percentile threshold was also used to reclassify predicted probabilities into BI hotspot areas (BI ≥ 5) to generate risk maps. Figure 4 and Figure S6 show the spatial distribution of BI hotspot areas at two different spatial scales during mosquito active periods in 2019 and 2020, specifically the months of June, July, and August.

Figure 4 illustrates the spatial distribution of BI hotspot areas at the township and 1000 m grid scale in 2019. Overall, the spatial distribution of BI hotspot areas in Guangzhou shows a predominant concentration in the southern and northern regions. However, significant differences are observed when comparing the spatial distribution of BI hotspot areas at the township scale. For example, the township of Tian He, located in the center of the city, is classified as a low-risk area for BI. However, the fine-scale risk map shows small parts of regions within it may experience larval infestation, categorizing them as hotspot areas with a high-risk for the DF epidemic. In contrast, only a few grids in Hengli Township are predicted as BI hotspot areas in June.

We calculate the area proportion of BI hotspot areas within each township to further clarify the differences at different spatial scales. Specifically, Figure S7 illustrates that Chi Ni township is classified as a BI hotspot areas; however, the proportion of BI hotspot areas within it is less than 3%. A similar finding is also observed in Dong Yong township, where the area proportion of BI hotspot areas is less than 50%. Additionally, the fine-scale predictions also indicated that BI hotspot areas are denser near the administrative boundaries of townships compared to other locations, practically in the southern regions of Guangzhou. In conclusion, (1) the fine-scale risk maps accurately reflect the overall spatial distribution characterization of BI hotspot areas in Guangzhou and show its significant heterogeneity within townships; and (2) regardless of whether a township is classified as a BI hotspot areas, the proportion of hotspot grids within the township is generally low.

## 4. Discussion

The fine-scale risk mapping method for the BI hotspot areas is validated using original vector surveillance data at the township scale and demonstrates high accuracy. A key strength of this study lies in the integration of data resampling techniques with the RF model, which significantly enhanced the model’s capacity to generate fine-scale risk maps at a 1000 m scale. Our findings indicate the following: (1) the integration of data resampling techniques with the RF model significantly improves model’s performance in downscaling BI hotspot areas to a finer scale; and (2) the fine-scale mosquito risk maps can effectively characterize the spatial heterogeneity of BI hotspot areas within townships. The fine-scale risk maps provide key evidence to support spatially targeted vector control efforts in intra-urban areas.

The choice of a suitable threshold, for variable selection and classifying probability of hotspot areas as well as defining high risk areas, plays a critical role in assessing the accuracy of predictive models. Firstly, we compared the model performance under two different thresholds for univariate variable selection: AUC = 0.5 and AUC = 0.6 [41,44]. Table S4 indicates that applying an AUC threshold of 0.5 for variable selection significantly improves model performance. An important reason for the decline in accuracy may be that the reduced data volume limits the data-driven approach’s ability to learn complex relationships among variables. Secondly, to analyze the importance of accurately predicting BI hotspot areas under the background of the DF epidemic, the 10th percentile probability cutoff method is selected to generate risk maps [44,53]. It is supposed achieves a balance between high recall and low specificity and introduces relatively small errors [52]. Previous studies used median or percentile value of mosquito density to classify high and low risk areas [14,39], while our study adopted a recommended threshold (i.e., BI = 5) to define the risk grades of BI. According to dengue outbreak prediction studies in Guangzhou [6,13], BI = 5 was found that as a critical threshold can efficient to identify dengue outbreaks in Guangzhou. Therefore, the threshold we used showed higher epidemiological values.

Data resampling technology is an effective strategy to address the problem of class imbalance [45]. Different from previous studies [54], our study focuses on the accuracy of predicting BI hotspot areas. Our results indicate that three resampling methods significantly improve the accuracy of predicting BI hotspot areas and the recall for optimal model is outperform than previous studies [55]. This finding is also consistent with the study of DF risk prediction in Guangzhou [31]. Notably, Table S5 reveals that the oversampling model exhibits substantial differences in performance metrics (e.g., ROC-AUC) between the training and test sets. The findings are also similar to previous studies and highlights overfitting as a common limitation of oversampling techniques [56,57,58]. We also recommends other researchers consider incorporating data resampling techniques to enhance predictive performance when the data is imbalanced.

A comprehensive evaluation of predictive model performance is crucial for obtaining spatial downscaling results on new test set. Table 2 and Table S2 show that the change range of AUC value in the prediction results is obviously smaller than the change in recall, regardless of whether single-year data or random sampling data are used as the test set. Several studies had previously found similar findings and highlighted significant limitations in the practical utility of ROC-AUC, particularly in cases of imbalanced data, where it exhibits lower sensitivity to changes in data distribution [49,59]. This shortcoming indicates that relying solely on the ROC-AUC metric for model evaluation is not applicable in all situations. Therefore, we additionally used three other metrics and, based on the evaluation criteria proposed in previous studies [6], completed the assessment of all predictive models’ performance. We also suggest that researchers should consider using more metrics rather than solely one general metric to evaluate model performance.

Fine-scale risk maps provide more benefits for local vector control efforts in guiding spatially targeted vector control. Community-involved vector control are widely used to reduce larval density, but the imprecise locating of hotspot areas often leads to resource waste [60]. On the one hand, with the help of fine-scale risk maps, decision-makers are capable of priority manage the BI hotspot areas within urban areas, such as urban village with high population density [10,25]. On the other hand, it breaks the constrain of administrative boundaries and suggests the BI hotspot areas are predominantly concentrated in areas where neat to townships boundaries. It is possible indicate that these regions are “lawless zones” and provide a favorable environment for larval development and adult mosquito reproduction [13,61]. Based on above advantage, we also recommend intensifying vector surveillance and control measures along administrative boundaries with the help of fine-scale risk maps.

This study has several limitations: (1) The spatial autocorrelation of BI across neighboring townships was not incorporated in this study. Neighboring townships with similar geographical environments may exhibit simultaneously high or low BI values, demonstrating significant spatial autocorrelation. However, some studies incorporated the spatial autocorrelation for mosquito-borne disease risk mapping, their results showing that the spatial autocorrelation had no significant impacts on prediction accuracy [62]. (2) Detailed information of intensity of vector control treatments was unavailable, so it is difficult to assess the whether different intensity level of vector control treatments could affect the identification of BI hotspot areas. Despite this limitation, we developed a model which incorporated a binary variable to assess the impact of vector control treatments on the prediction of BI hotspot areas. Figure S7 shows that explicit expression vector intervention measures have limited improvement on the prediction accuracy of BI hotspot areas. One possible reason is that the location of BI hotspot areas in Guangzhou is relatively stable [63]. (3) This study used vector surveillance data from Guangzhou city over two consecutive years to build predictive models, representing a relatively small dataset. Nevertheless, we established prediction models using biweekly monitoring data and monthly monitoring data, respectively, and found that increasing the data volume has no significant improvement on the prediction accuracy of the BI hotspot areas (Table S3).

## 5. Conclusions

In conclusion, this study takes Guangzhou and *Ae. albopictus* as examples to provide a fine-scale (1000 m) risk mapping method of hotspot areas by integrating a data resampling technique and fine-scale input data. Fine-scale risk maps provide tools for policymakers to guide a pixel-level targeted vector control and design improved vector surveillance in intra-urban areas. We conjecture that the proposed fine-scale risk mapping method might be used for the large-scale prediction of mosquito hotspot areas in other locations and other vector-borne diseases, including DF.

## Figures and Tables

**Table 1:** Environmental and socio-economic variables.

Variables	Temporal Resolution	Spatial Resolution	Sources
Mean temperature	Monthly	1000 m	https://data.tpdc.ac.cn/ (accessed on 24 June 2025).
Cumulative rainfall	Monthly	1000 m	https://data.tpdc.ac.cn/ (accessed on 24 June 2025).
Mean NDVI	Monthly	10 m	https://developers.google.com/earth-engine/datasets/ (accessed on 24 June 2025).
SHEI	Year	2.4 m	http://geoscape.pku.edu.cn/dataproject.html (accessed on 24 June 2025).
Population densities	Year	100 m	https://hub.worldpop.org/ (accessed on 24 June 2025).

**Table 2:** Accuracy evaluation results of four RF models.

Models	ROC-AUC	Specificity	Recall	G-Means
Model1	0.8643	0.9892	0.2469	0.4911
Model2 *	0.8468	0.7682	0.7977	0.7821
Model3	0.8614	0.9689	0.3903	0.6124
Model4	0.8609	0.9396	0.5604	0.7244

* It is the optimal predictive model. Model1 represents the original RF model, Model2 is the RF model processed by undersampling techniques, Model3 is the RF model processed by oversampling techniques, and Model4 is the RF model processed by hybrid sampling techniques.

## Data Availability

The data presented in this study are available on request from the corresponding author.

## Supplementary Materials

The following supporting information can be downloaded at: https://www.mdpi.com/article/10.3390/insects16070661/s1. Figure S1: The single AUC of each variables; Figure S2: Pearson’s correlation matrix between this variables; Figure S3: Spearman’s rank correlation matrix between this variables; Figure S4: Prediction accuracy under both vector intervention and without intervention conditions; Figure S5: The list for several township; Figure S6: Risk map at township scale for Guangzhou 2020; Figure S7: The area percentage for high-risk grid within township scale; Table S1: The prediction accuracy of the test set using average rainfall as predictor variables; Table S2: The prediction accuracy of test set in 2020; Table S3: The prediction accuracy of test set using biweekly surveillance data; Table S4: Model performance of variable selection results when the threshold of AUC is 0.6; Table S5: Model performance in ROC-AUC metrics under train and test dataset.
