# Supplementary material for: Fine-Scale Risk Mapping for Dengue Vector Using Spatial Downscaling in Intra-Urban Areas of Guangzhou, China
Source: Insects. 2025 Jun 25;16(7):661. doi: 10.3390/insects16070661 (PMC12295946; doi:10.3390/insects16070661)
Supplement: Supplementary file 1 [file insects-16-00661-s001.zip › insects-3550124-supplementary.pdf]

Supplementary Material

Fine-scale risk mapping for dengue vector using spatial downscaling in intra-urban area of Guangzhou, China

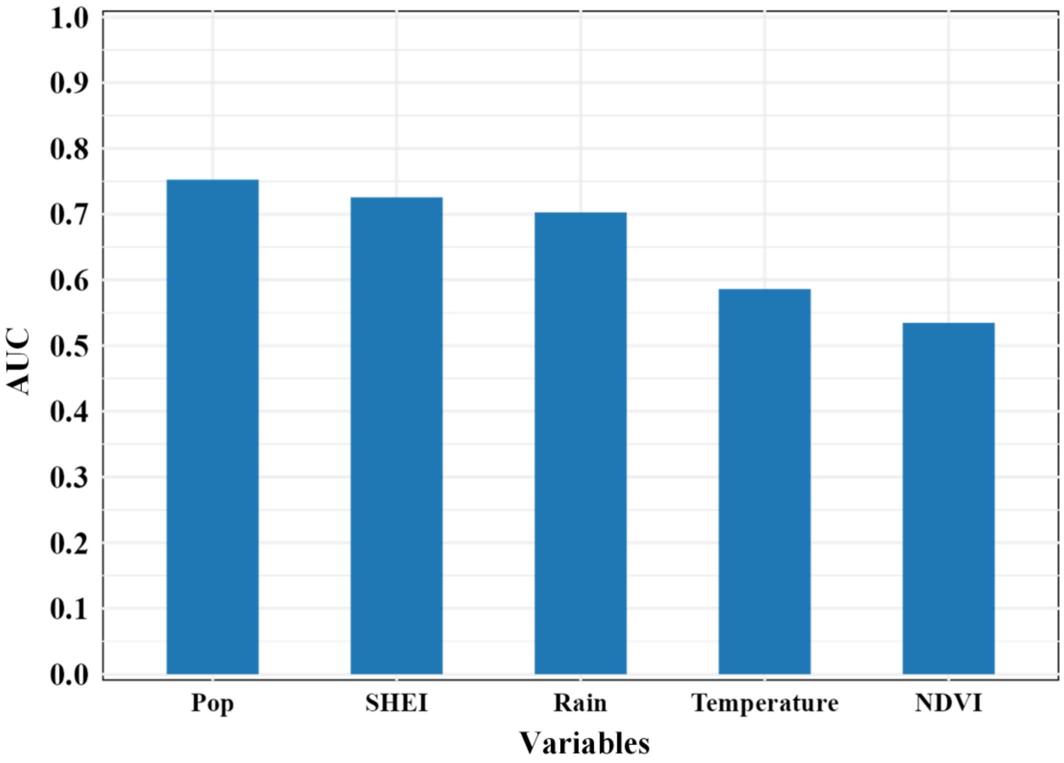

Figure S1： Univariate analysis results between environmental and socio-economical variables and larval density levels. Abbreviations: Pop, Population density; SHEI, Shannno eveness index; NDVI, Normalized difference vegetation index; Temp, Mean temperature; Rain, Cumulative rainfall

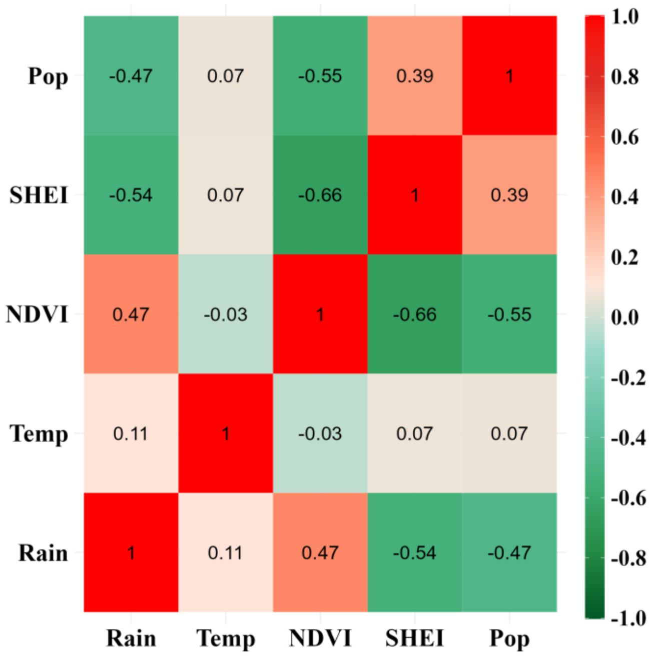

Figure S2: Pearson correlation coefficient matrix among environmental and socio-economical variables. Abbreviations:Pop,Population density;SHEI, Shannno eveness index; NDVI,Normalized difference vegetation index; Temp, Mean temperature; Rain, Cumulative rainfall

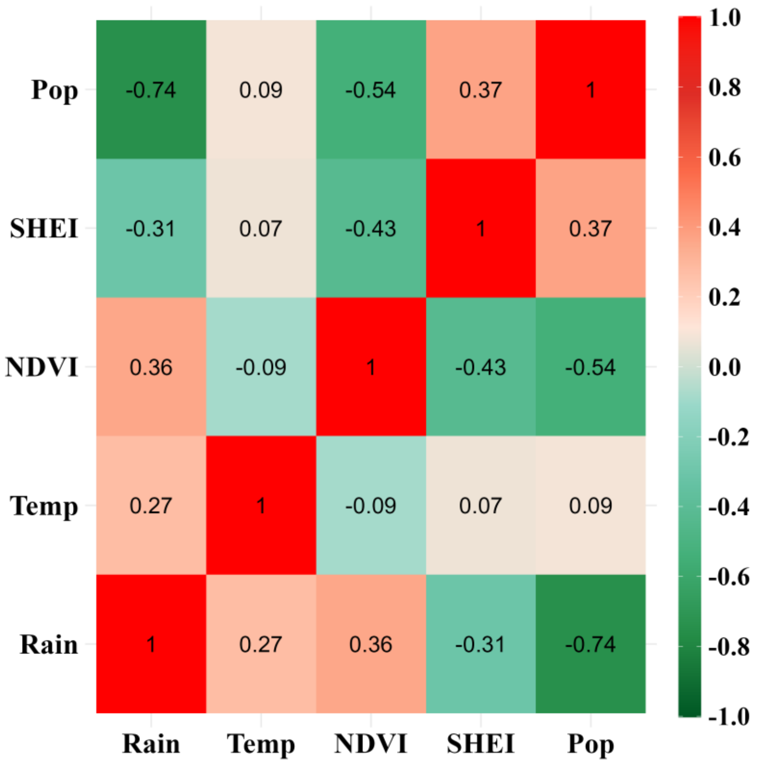

Figure S3: Spearman's rank correlation coefficient matrix among environmental and socio-economical variables. Abbreviations:Pop,Population density;SHEI, Shannno eveness index; NDVI,Normalized difference vegetation index; Temp, Mean temperature; Rain, Cumulative rainfall

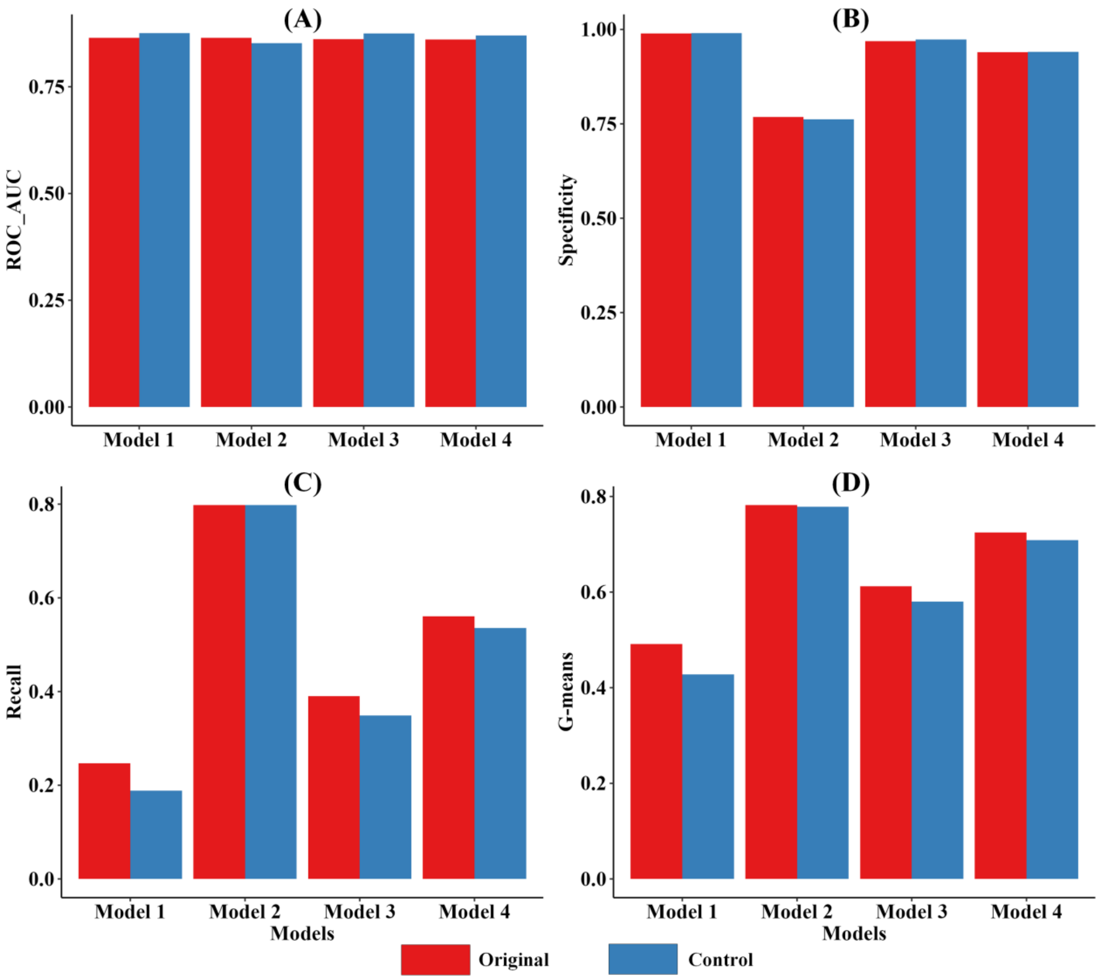

Figure S4: Prediction accuracy of the test set when models are developed without considering intervention and under intervention conditions. The label of original in the figure indicates that the effect of vector control treatments without consideration in the modeling process; The label of control in the figure indicates that vector control treatments are implemented from March to November.

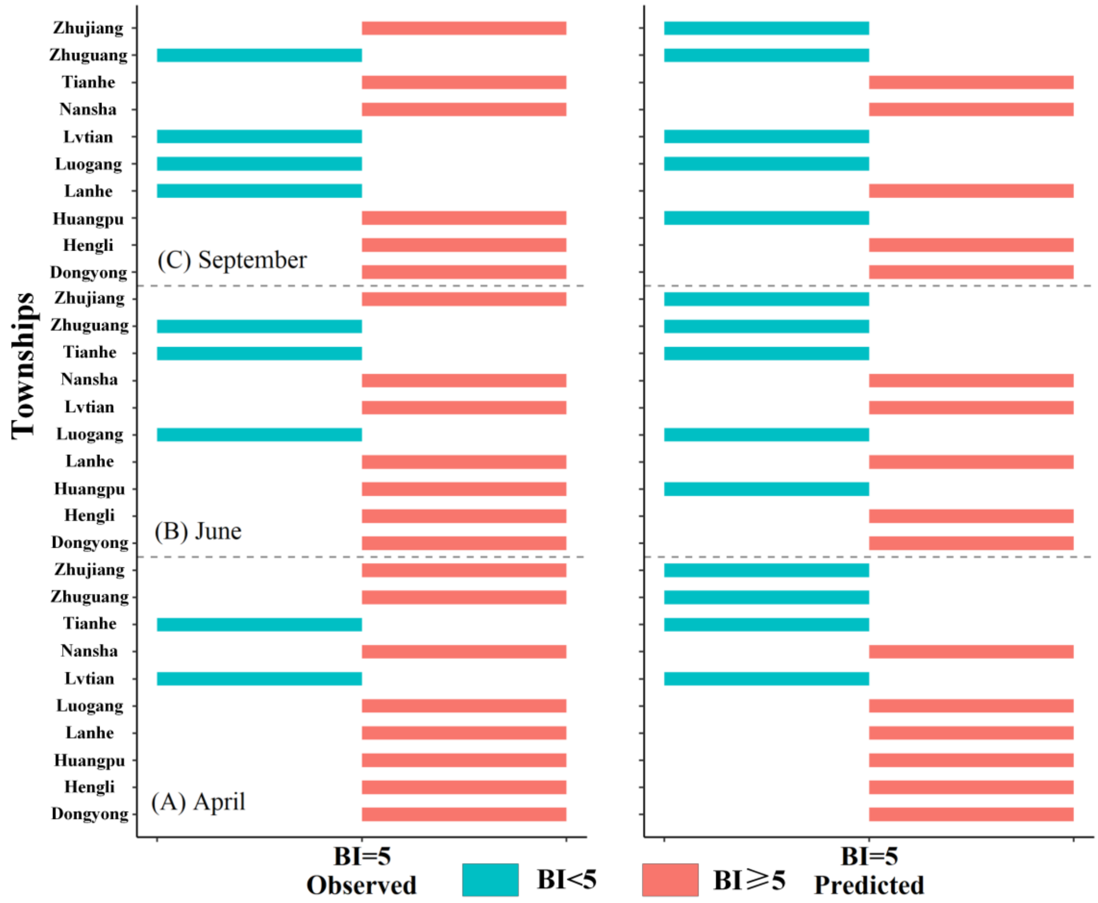

Figure S5: Compared the observed and predicted results for several specific townships of Guangzhou in 2019.

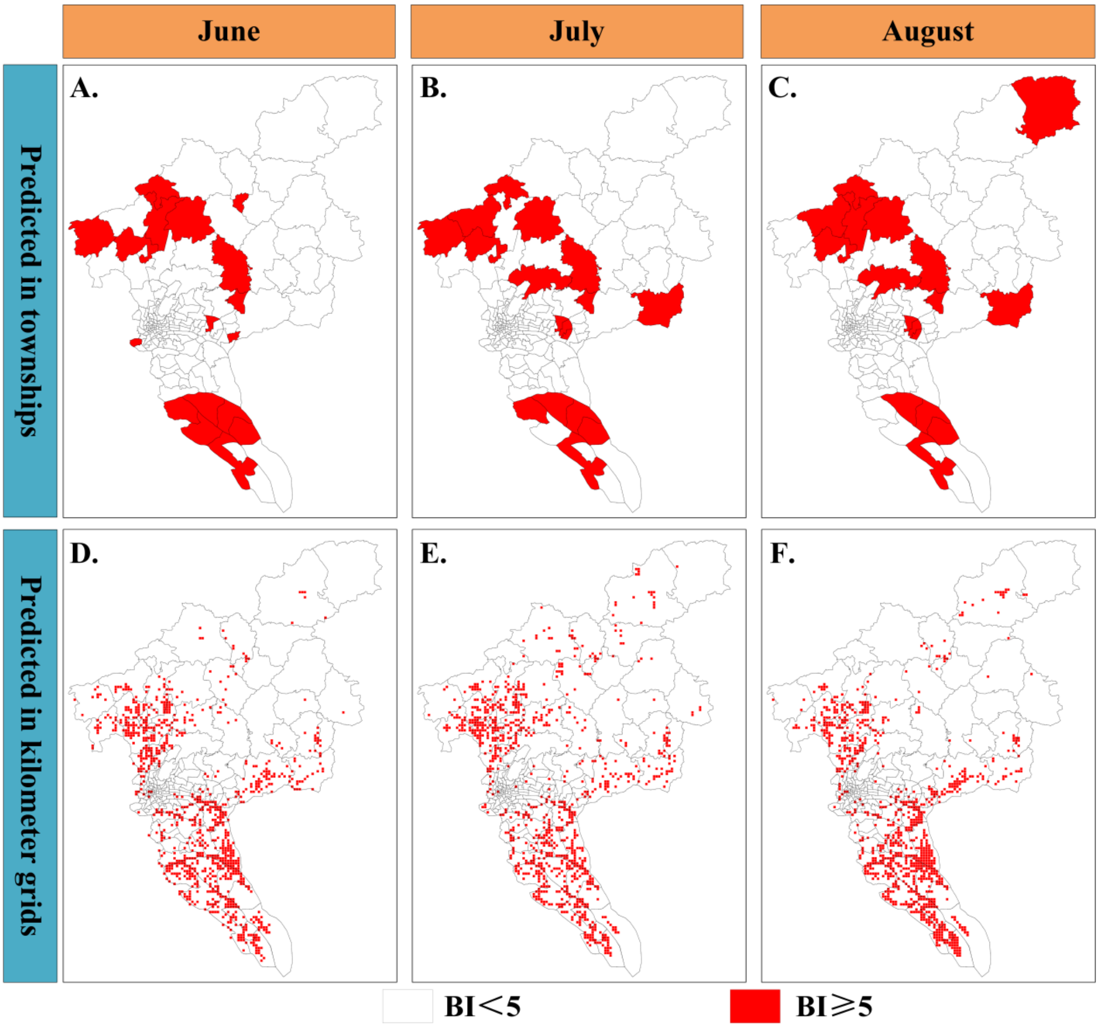

Figure S6: Mosquitoes risk map for township and 1000 m grid scale at 2020.

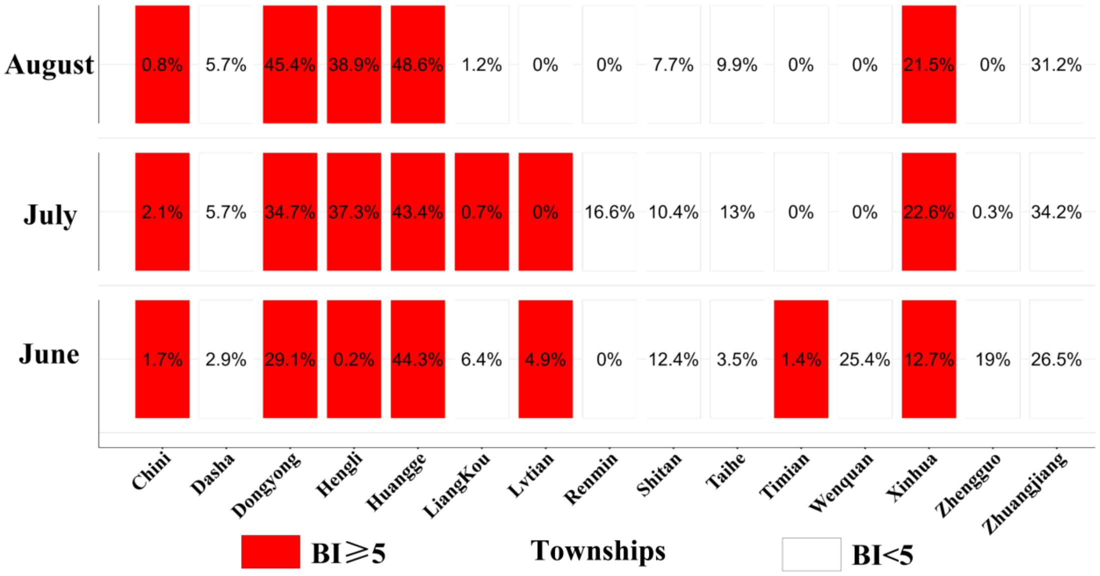

Figure S7: The area proportion of hotspot areas within townships. The numeric within a rectangle represent the value of area proportion of hotspot areas.

Table S1. The prediction accuracy of the test set when using average rainfall and cumulative rainfall as predictor variables, respectively.

Models	ROC-AUC	Specificity	Recall	G-means
Average	0.8528	0.7706	0.7947	0.7814
Cumulative	0.8468	0.7682	0.7977	0.7821

Average indicates the average rainfall as the predictor variable. Cumulative indicates the cumulative rainfall as the predictor variable.

Table S2. The prediction accuracy of test set under two different verified methods.

Models	ROC-AUC	Specificity	Recall	G-means
Method1	0.7995	0.7341	0.7420	0.7377
Mothod2	0.8468	0.7682	0.7977	0.7821

Method 1 indicates using the data of 2020 as the test set to verify model's performance. Method 2 indicates that 20% of the total data is randomly used as the test set to verify model's performance.

Table S3. Comparison of prediction accuracy using biweekly vector surveillance data versus monthly average vector surveillance data

Models	ROC-AUC	Specificity	Recall	G-means
Model1	0.8617(-0.0026)	0.9658(-0.0234)	0.3896(0.1400)	0.6120(0.1209)
Model2*	0.8450(-0.0012)	0.7707(+0.0025)	0.7654(-0.0323)	0.7674(-0.0147)
Model3	0.8697(+0.0083)	0.9495(-0.0194)	0.4058(0.0155)	0.6780(0.0656)
Model4	0.8586(-0.0014)	0.9206(-0.019)	0.5789(0.0185)	0.7292(0.0048)

* It is the optimal predictive model. Model1 represent the original RF, Model2 is the RF processed by undersampling techniques, Model3 is the RF processed by oversampling techniques and Model4 is the RF processed by hybrid sampling techniques. Value in parentheses indicate the the magnitude of changes compared to the monthly BI data modeling results.

Table S4. Model performance of variable selection results when AUC is equal to 0.6.

**Models**	**ROC-AUC**	**Specificity**	**Recall**	**G-means**
Model1	0.8278(-4.2%)	0.9783(-1.1%)	0.1883(-23.7%)	0.4286(-12.7%)
Model2*	0.8076 (-4.6%)	0.7674(-0.01%)	0.6883(-13.7%)	0.7264(-7.1%)
Model3	0.8254(-4.2%)	0.9419(-2.9%)	0.3474(-11.0%)	0.5713(-6.7%)
Model4	0.8251(-4.2%)	0.9143(-2.7%)	0.4645(-17.1%)	0.6512(-10.0%)

* It is the optimal predictive model. Model1 represent the original RF, Model2 is the RF processed by undersampling techniques, Model3 is the RF processed by oversampling techniques and Model4 is the RF processed by hybrid sampling techniques. Value in parentheses indicate the percentage decrease in prediction compared to when AUC=0.5 was

used as a threshold.

Table S5. Model performance in ROC-AUC metrics under train and test data set.

Models	Train data set	Test data set
Model1	0.9317	0.8643
Model2	0.9213	0.8468
Model3	0.9960	0.8614
Model4	0.9861	0.8609

Model1 represent the original RF, Model2 is the RF processed by undersampling techniques, Model3 is the RF processed by oversampling techniques and Model4 is the RF processed by hybrid sampling techniques.
